# Segmentation of the lateral femoral notch sign with MRI using a new measurement technique

**DOI:** 10.1186/s12891-015-0677-0

**Published:** 2015-08-21

**Authors:** Thomas Hoffelner, Isabel Pichler, Philipp Moroder, Michael Osti, Martin Hudelmaier, Wolfgang Wirth, Herbert Resch, Alexander Auffarth

**Affiliations:** Department of Traumatology, Sports Injuries of the Paracelsus Medical University, Salzburg, Austria; Institute of Anatomy and Musculoskeletal Research of the Paracelsus Medical University, Salzburg, Austria; Department of Traumatology, Sports Injuries of the University of Feldkirch, Voralrberg, Austria

## Abstract

**Background:**

The goal of this present study was to precisely determine the dimension and location of the impaction fracture on the lateral femoral condyle in patients with an ACL rupture.

**Methods:**

All patients with post-injury bi-plane radiographs and MRI images after sustaining a tear to the anterior cruciate ligament were included. Lateral radiographs of the affected knee were inspected for a lateral femoral notch sign. MRIs of patients with a lateral condylopatellar sulcus ≥1.5 mm were used to segment and measure the lateral condylopatellar sulcus. The MRI examination was interpreted by an expert in musculoskeletal radiology. The study was approved by the ethics committee of the state of Salzburg.

**Results:**

A “lateral femoral notch sign”was seen in 50 patients. The average total surface area of the lateral femoral condyle was 3271.7 mm^2^ (SD 739.5 mm^2^). The defect had a mean surface area of 266.1 mm^2^ (SD 125.5 mm^2^), a mean volume of 456.5 mm^3^ (SD 278.5 mm^3^), a mean depth of 3.0 mm (SD 0.8 mm). On average 169 mm^2^ (SD 99.6 mm^2^) of the surface of the condyle were affected by the impaction fracture which corresponds to 5.2 % (SD 2.8 %) of the surface of the lateral femoral condyle. In 51 % the impaction fracture was located in the central-external area of the femoral condyle.

**Conclusions:**

In cases of a clinically suspected ACL rupture lateral radiographs of the knee should be checked for a lateral femoral notch sign further MRI for confirmation should be performed. Knowing of the precise defect on the lateral femoral condyle is an additionally valuable information, as concomitant injuries to a rupture of the anterior cruciate ligament increase the risk for early-onset osteoarthritis in the future.

## Background

It is known that 80–85 % of all osseous injuries observed in the knee after a tear of the anterior cruciate ligament (ACL) are found in the lateral compartment [1–3]. The injury mechanism that leads to an ACL tear involves an anterior subluxation of the tibia in relation to the femur. This can provoke a collision between the lateral femoral condyle and the postero-lateral edge of the tibial plateau, [4, 5] which may result in a “kissing contusion”. The location of this engagement depends on the degree of flexion of the knee while subluxating. The more forceful such an impact is, the greater the damage to the lateral femoral condyle may be [6]. In most cases the lateral femoral notch has only radiographic significance with no need for surgical treatment. These lesions like bone bruises may only be visible with magnetic resonance imaging (MRI), while in other cases an impaction of the subchondral cortical bone may be apparent on a conventional X-ray image. These highly developed injuries have a clinical relevance due to deformation of the articular surface of the femoral condyle, as a possible precursor of osteoarthritis [7]. Such osseous injuries with impaction fracture of the lateral femoral condyle are referred to as the lateral femoral notch sign. These have been reported to accompany ruptures of the ACL in 20–60 % [4, 7, 12]. Apart from being an sign for the presence of a torn ACL, [8] these impaction fractures can also be relevant due to their long-term detrimental effect to the joint [9, 10]. As knees with abnormal notches on the femoral condyle showed lateral meniscus injuries more frequently than those without such notches [11]. Cobby et al. reported that patients with an intact ACL showed an average depth of the sulcus of 0.5 mm while patients with a torn ACL showed an average sulcus depth of 0.9 mm. The authors concluded that a sulcus depth exceeding 1.5 mm which corresponds to a standard deviation 3 times above the norm could be considered as a reliable indicator of an ACL rupture, a cut-off, which was also chosen in this investigation to define patients for further investigation of their MRI [8]. In most cases, this finding may not be clinically relevant in terms of the need for a surgical repair. Although very little literature exits, it seems that more extensive impactions that lead to a relevant deformation of the articular surface of the femoral condyle can be associated with an increased risk of early-onset osteoarthritis [7]. Attempts have been made to treat extensive osteochondral impaction fractures of the lateral femoral condyle, in order to prevent long-term consequences such as osteoarthritis [7, 12, 13]. For this reason several authors tried to characterize the localization of this radiologic sign. Kaplan et al. reported in their study, that the bone bruise would be located in the central to anterior part of the lateral femoral condyle, but they had not divided the condyle into sub-areas [14]. Speer et al. investigated sagittal and coronal MRIs in order to define the precise localization of the impaction fracture on the lateral femoral condyle. They reported that the lesions were predominantly located in the area of the sulcus terminalis and obviously would tend to be located laterally rather than medially [15]. Graf et al. were the first group who divided the lateral femur in defined regions for a better specification of the location [1]. Therefore the goal of this present study was to precisely determine the size and location of impaction fractures on the lateral femoral condyle (“lateral femoral notch sign”) in patients with an ACL rupture.

## Methods

**Fig. 1:**
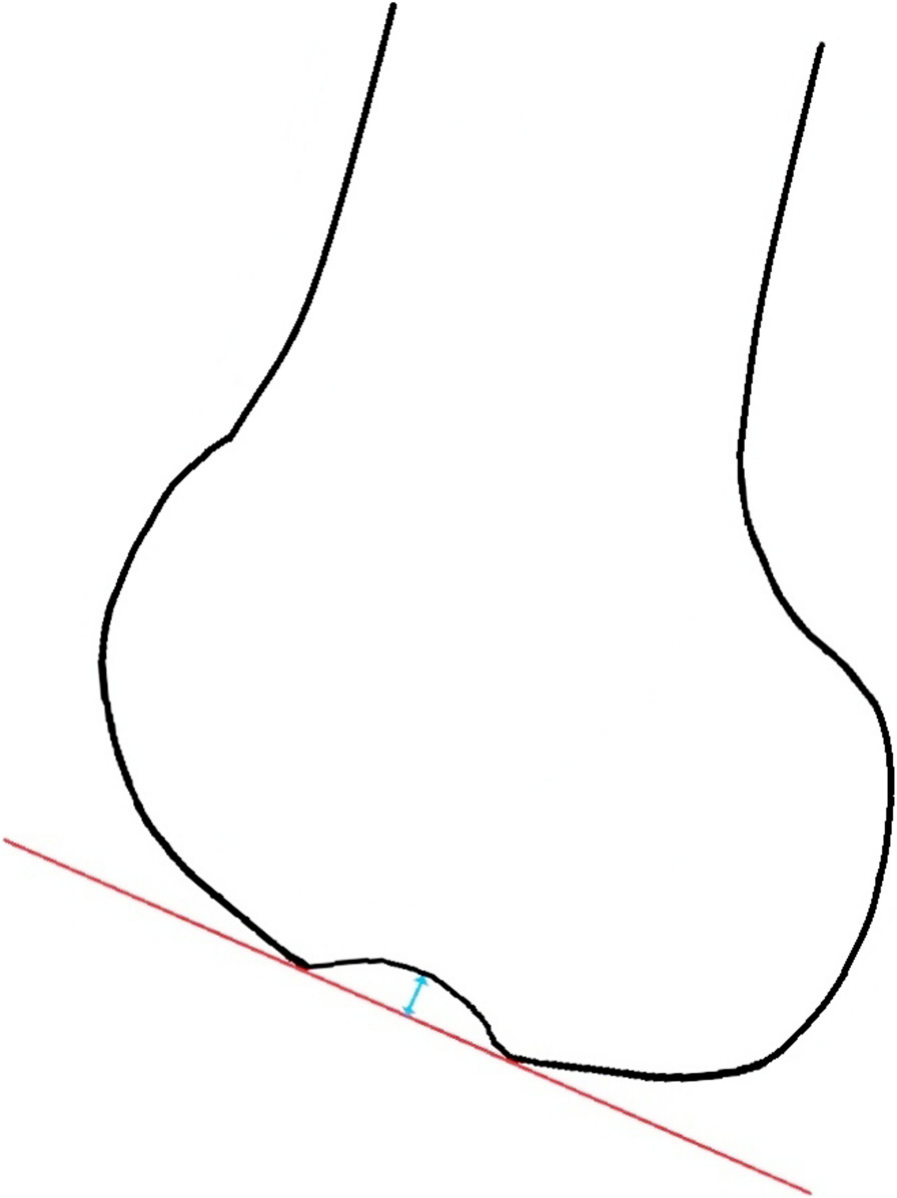
Measurement technique to determine the depth of the femoral notch on lateral x-rays

The knee database of the Department of Traumatology and Sports Injuries was searched for patients who had an arthroscopy of the knee between 2006 and 2010 independent of the preoperative diagnosis. Next, all surgical reports were reviewed, and cases with pathology of the ACL were selected for further investigation. Patients originally scheduled for meniscal surgery, as well as for an ACL repair were included. All of this patients underwent an examination with MRI. This left 422 patients for radiological examination. Of these 20 (4.7 %) previously undergo ACL replacement, 310 (73.5 %) had a complete tear of the ACL, 4 (0.9 %) had an osseous avulsion of the ACL, 63 (14.9 %) had a partial tear and 25 (5.9 %) had an elongation of the ACL. All x-rays of these 422 patients were retrospectively examined for an unusually deep lateral condylopatellar sulcus by employing the measurement technique proposed by Warren et al [8]. All lateral x-rays of the knee were performed in a standardized technique by our radiologist assistant. Afterwards the depth of the lateral condylopatellar sulcus was measured by drawing a tangential line across the sulcus on the articular surface of the lateral femoral condyle. Then the depth of the sulcus was measured perpendicular to that line at its deepest point [16] (Fig. 1). This revealed 64 knees with a sulcus depth over 1.5 mm that were selected for further investigation. Reviewing these MRI, 14 with a suspicious x-ray apparently had no evidence of a lateral femoral notch sign, so they were excluded from further investigation, which left 50 knees for MRI evaluation. All images were done for patient examination and corresponds to normal clinical routine, yet written consent were obtained of all of the patients. The study was approved by the ethics committee of the state of Salzburg, Sebastian-Stief-Gasse 2, A-5010 Salzburg, Austria in terms of stating that an appraisal would not be necessary, because no harm to the patients performing an MRI of the knee would be expected.

### MRI evaluation

The MRI were obtained from diagnostic routines of the patients, using Siemens (Eschborn, Germany) and Philips Medical Systems (Andover, USA) scanners with magnetic field strengths between 1.0 and 3.0 Tesla. As clinical routines do not include specific sequences for cartilage quantification, standard diagnostic sequences with good cartilage delineation were chosen. If available, proton density weighted turbo spin echo sequences were preferred, but plain turbo spin echo or spin echo sequences were also accepted (Table 1). All images were segmented at the university’s institution of anatomy using a proprietary software suite (Chondrometrics GmbH, Ainring, Germany).

**Table 1:** MR sequences used for data acquisition in 51 subjects. Please note that similar sequences are grouped, and ranges are given for MR parameters which vary slightly within the group

Cases	Vendor	Scanner model	Sequence	Coil	Field	Slice Sp	Slice Th	IPR	TR	TE	FA
					[T]	[mm]	[mm]	[mm^2^]	[ms]	[ms]	[°]
19	Siemens	Symphony	PD + t2 TSE	Ex	1.50	4.8	4.0	0.469-0.547	3300	15	150
5	Siemens	Symphony	PD TSE	Ex	1.50	4.8	4.0	0.293-0.313	2000	13	150
1	Siemens	Symphony	TSE	Ex	1.50	4.8	4.0	0.469	525	13	180
2	Siemens	Harmony			0.95	0.8	4.0	0.313	715	20	90
2	Siemens	Avanto	SE	B	1.49	4.4	4.0	0.375	408	11	150
1	Siemens	Vision	PD TSE	Ex	1.50	4.4	3.5	0.625	2010	35	170
7	Siemens	Vision	PD TSE	Ex	1.50	4.6	3.5	0.313-0.379	18302-2110	12-23	170
1	Siemens	Verio	TSE	Kn	3.00	4.4	4.0	0.442	500	12	150
1	Philips	Achieva	TSE	B	3.00	3.3	3.0	0.313	509	20	90
6	Philips	Intera	PD	B	1.00	3.3	3.0	0.332	1887-2230	35	90
1	Philips	Intera	PD	B	1.00	3.3	3.0	0.391	1300	25	90
1	Philips	Intera	PD	B	1.50	3.7	3.4	0.703	660	18	35
4	Philips	NT Intera	PD	B	1.00	3.3-3.4	3.0-3.1	0.351	1691-1696	35	90

*Slice Sp* slice spacing, *Slice Th* Slice thickness, *IPR* in plane resolution, *TR* time of repetition, *TE* time of echo, *FA* flip angle, *PD + t2* Proton density plus t2 weighted, *TSE* turbo spin echo, *SE* spin echo, *Ex* extremity coil, *B* body coil, *Kn* knee coil

### Segmentation of the MRI

**Fig. 2:**
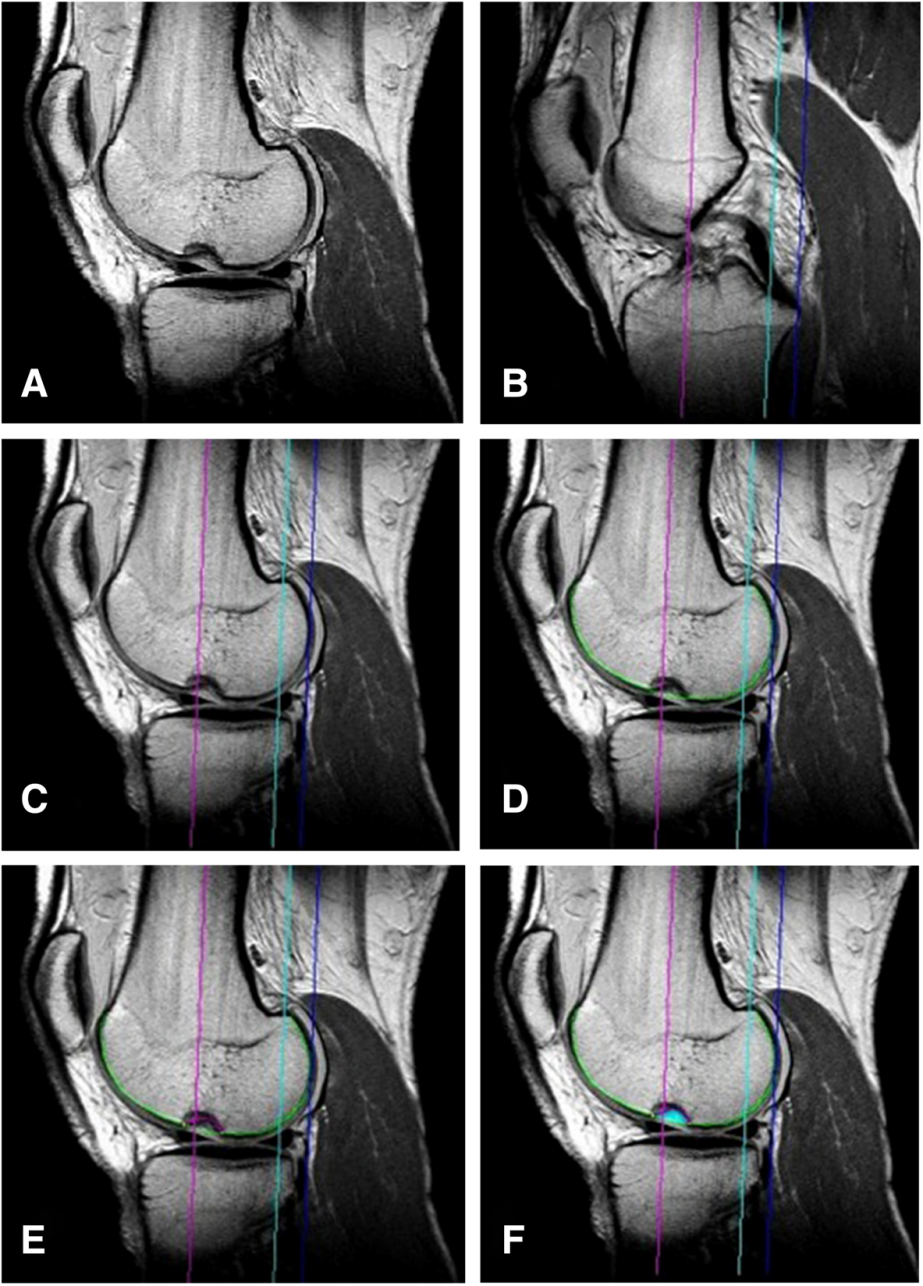
Segmentation of the MRI. (**a**) sagittal MRI with lateral femoral notch. Alignment of the axis: (**b**) line parallel to the axis of the femoral shaft running through the deepest point of the intercondylar notch (pink line), (**c**) most posterior aspect of the lateral femoral condyle (dark blue line), “weight bearing area” (light blue line), both parallel to the axis of the femoral shaft. (**d**)Segmentation of the defect: the subchondral bone area (green line), (**e**) marking of the defect (curved pink line), (**f**) area of the defect (light blue area)

The MRI examination was interpreted by an expert in musculoskeletal radiology. The measurement based on a technique described by Dr. Eckstein [11, 17]. Segmentation of the entire femoral subchondral bone surface area was performed manually in all slices between the trochlear notch of the femur and the most lateral aspect of the lateral femoral condyle. In the region affected by the impaction fracture, the gap was bridged by a contour approximating the contour of the subchondral bone area under normal anatomical conditions. The depressed portion was separately marked (Fig. 2 d-f). Additionally a 3D reconstruction of the subchondral bone area and the impaction fracture was performed from the segmentations.

The sagittal MRI of the subchondral bone area was divided into three segments in anterior-posterior direction: A plane constructed from a) the vector through the trochlear notch and parallel to the femoral shaft and from b) the vector connecting the most posterior points of the medial and lateral femoral condyle was used to separate the trochlea of the femur from the lateral femoral condyle. A second plane was positioned at 60 % of the distance between the trochlear notch and the most posterior points of the femoral condyles and parallel to the 1st plane to separate the central, weight-bearing segment of the lateral femoral condyle from the posterior segment of the lateral femoral condyle. The trochlea of the femur, the central segment of the femoral condyle, and the posterior segment of the femoral condyle were additionally separated into one internal and one external segment by using 50 % of the maximum medial to lateral extent of the segmented subchondral bone area as criterion (Fig. 2a-c).

The extent of the lateral femoral notch was determined by different parameters. The maximum extension of the defect in the antero-posterior direction, its transverse extension, as well as the defects depth were measured in absolute values (millimeters). The primary articular surface area (Fig. 2d, green line) affected by the defect was measured in square centimeters and was related to the surface of the whole lateral condyle in percent. Additionally the concave surface (Fig. 2e, pink line) area of the lateral femoral notch was determined in absolute values (square centimeters) and finally the volume of the defect was calculated in cubic millimeters.

## Results

### Mapping of the lateral femoral condyle

**Fig. 3:**
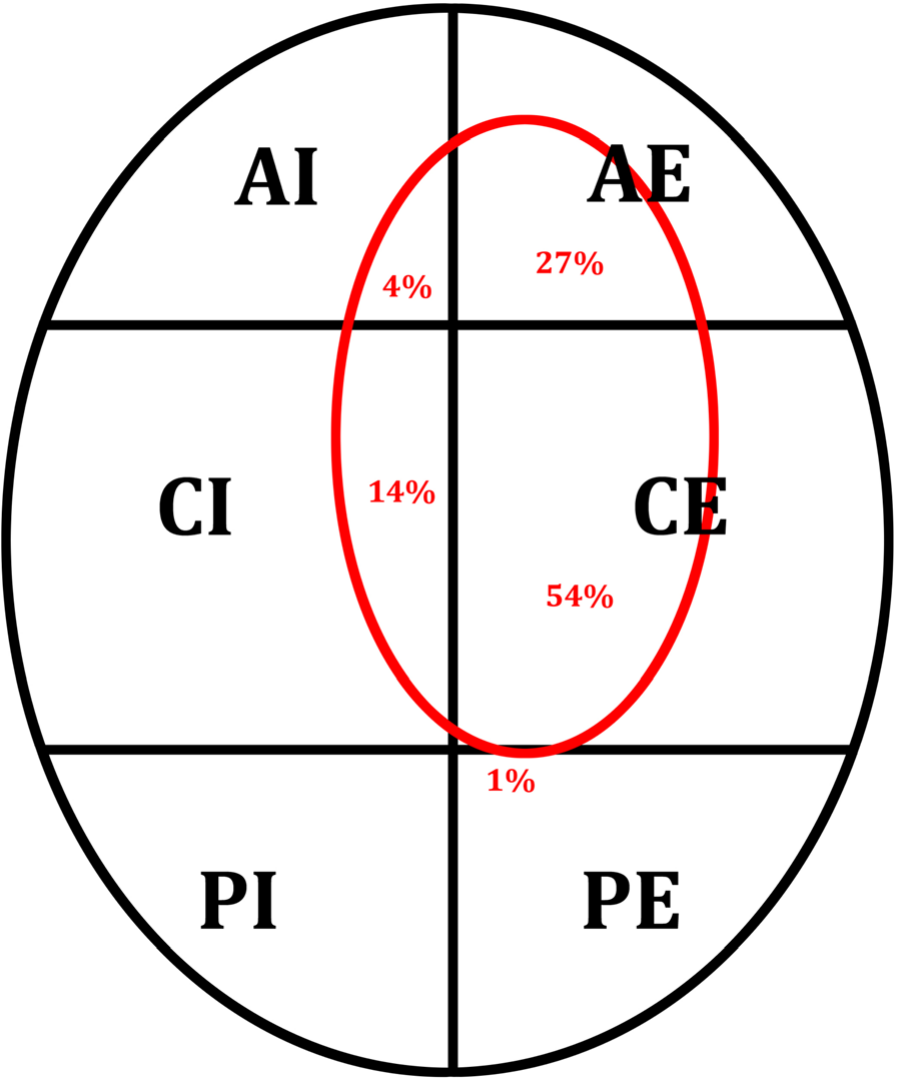
Schematic figure of the segmentation of the lateral femoral condyle in six segments (2D, transversal): anterior-internal (AI), anterior-external (AE), central-internal (CI), central-external (CE), posterior-internal (PI), posterior-external (PE). Internal correlates to the medial aspect, external to the lateral aspect of the condyle. The red circle illustrates the average distribution of the affected areas of the lateral femoral condyle

Most often, osseous impactions were located in the central internal and external segments which were referred to as the main weight bearing area. Sixty-eight percent affected by the impaction, this equaled 30.8 % of the defined weight bearing area, were located in the central internal and external regions of the condyle’s surface. An overview of the distribution of the defect surface affecting the defined segments of the lateral femoral condyle’s surface is given in Fig. 3.

### Magnetic resonance imaging results

The lateral femoral condyle had an average surface area of 32.7 cm^2^ (SD 7.4 cm^2^). The fracture had a mean surface area of 2.7 cm^2^ (SD 1.3 cm^2^), a mean volume of 456.5 mm^3^ (SD 278.5 mm^3^), and a mean depth of 3.0 mm (SD 0.8 mm). The average area affected by the impaction fracture was 1.7 cm^2^ (SD 0.1 cm^2^) or 5.2 % of the lateral femoral condyle surface area. The variables calculated for the six individual segments are displayed in Table 2.

**Table 2:** Average values calculated for the six regions the lateral femoral condyle was divided in

Region^1^	Lateral condyle’s surface^2^	Length of the defect^3^ (a.p.^5^)	Width of the defect^3^ (m.l.^6^)	Depth of the defect^3^	Affected total surface^2^ area	Percent of region’s surface^2^	Concave surface of defect^2^	volume of defect^4^
Central Extern	4.0	10.7	13.0	2.8	0.9	0.3	1.3	221.0
Central Intern	3.2	8.6	4.7	2.2	0.2	0.08	0.5	85.3
Anterior Extern	6.6	7.3	11.0	2.1	0.5	0.07	0.7	120.8
Anterior Intern	8.4	3.4	3.3	1.5	0.06	0.008	0.2	28.2
Posterior Extern	6.8	0.1	0.2	0.1	0.004	0.001	0.007	1.3
Posterior Intern	3.7	0.0	0.0	0.0	0.0	0.0	0.0	0.0

^1^as described in Fig. 3

^2^square centimeters (cm^2^)

^3^millimeters (mm)

^4^cubic millileters (mm^3^)

^5^a.p. (antero-posterior)

^6^m.l. (medio-lateral)

## Discussion

To our knowledge this study is the first to provide a complete analysis of the extension, volume, depth and localization of the lateral femoral notch sign using MRI. Bone bruises or micro-fractures are frequent concomitant lesions of ACL ruptures but are mostly only detected on MRI [14, 15, 18]. Depending on the severity of the injury, lesions vary between mere bone bruises and true impaction-fractures of the lateral femoral condyle, the so called “lateral femoral notch sign” [7].

This study has several limitations. A main issue is the large number of scanners and MR sequences with differing MR parameters (i.e.: resolution, echo time, flip angle) employed. However, it has been shown that the precision of the technique is excellent, [19]. and that deviations and variations in MR sequence parameters affect the results of cartilage quantification less than 10 % [20]. Finally, this is a retrospective study which can only use the available materials. First of all it is difficult to distinguish between a deep lateral condylopatellar sulcus and a small impaction-fracture. The depth of an individual’s native sulcus is impossible to determine, and evaluation of the contralateral uninjured knee may be necessary to obtain a surrogate measure for the depth of the uninjured sulcus, assuming that the patient is symmetric. A distortion of the results might have been caused by the fact that the analyzed lateral femoral condyles respectively the condylopatellar sulci were convex, but under physiologic conditions this convex shape can sometimes also show a small pit. Therefore, the depth of the lesions might have been overestimated as the initial sulcus depth was unknown, but according to the work of Cobby we estimated that the mean depth in patients without a tear of the ACL is 0.45 mm [16]. Still, all MRI reviewed were those, which had been acquired in the wake of the injury. A final limitation is the varying quality of the investigated MRI, which though reflects daily routine in an outpatient clinic. Nevertheless the resolution of the MRI images has been high enough in all cases, that the defects have been segmented with a high amount of voxel, that the precision most probably did not suffer from [21].

Graf et al. were the first group who divided the lateral femur in defined regions for a better specification of the location. With the knee in full extension, they defined the anterior part as the part in front of the anterior horn of the lateral meniscus, and the posterior part as the part posterior to the posterior horn of the meniscus [18]. In their study population the majority of the lesions were observed in the middle part of the lateral femoral condyle, which resembles our findings. Warren et al [8]. and Cobby et al. [16] compared X-rays of patients with and without a rupture of the ACL. Warren et al. observed a sulcus of 1.5 mm or deeper in 2 patients out of 52 with acute rupture of the ACL and in 13 out of 101 patients with a chronic rupture of the ACL. In comparison, only 1 patient out of 47 with an intact ACL showed a sulcus deeper than 1.0 mm. Warren et al. concluded that a sulcus sign more than 2.0 mm in depth detected on a X-ray image would be rare, but reliable indicated an ACL rupture [8]. In a study by Cobby et al. 62 patients without and 41 patients with a verified rupture of the ACL were examined. The authors concluded that a lateral condylopatellar sulcus exceeding 1.5 mm depth, which corresponds to a standard deviation 3 times above the norm, would be a strong indicator for a sustained ACL injury [16]. Comparing our findings to the work of Warren et al. [8] and Cobby et al. it is obvious, that the size of the lateral femoral notch helps to determine the presence of a ruptured ACL. In this study 4 patients only with a partial rupture of the ACL also showed a lateral femoral notch sign. This finding leads us to the opinion that injuries which only cause a partial rupture of the ACL, also seem to exert significant force to the articular joint in some specific cases. The long-term success of an ACL reconstruction not only depends on restoring knee stability and function but also on preventing degenerative changes [22]. According to the knee trauma cascade [23] concomitant injuries to a rupture of the anterior cruciate ligament increase the risk for early-onset osteoarthritis in the future. In most cases the lateral femoral notch may only radiographically picture a significant lesion with no need for surgical treatment, whereas large defects can become clinically relevant. Because of the deformed articular surface in weight-bearing areas of the femorotibial joint, early-onset osteoarthritis may result on the long term, if such lesions are left untreated [7, 12, 13]. These long-term consequences may be influenced by several factors such as apoptosis of chondrocytes, loss of proteoglycans, and destruction of collagen structures [24]. Furthermore it is possible that such subchondral osseous injuries may heal with callus-formations that may thicken and stiffen the subchondral bone layer. The resulting diminished flexibility of the subchondral bone can lead to increased pressure applied to the overlying cartilage leading to early-onset osteoarthritis in the long-term [25]. What our study showed is, that unfortunately that the lesion is in most of the cases in the weight bearing area (CI/CE) of the femoral condyle. According to Costa-Paz et al. there is reason to believe that the impaction fracture of the lateral femoral condyle is non-transient [25]. Therefore knowing the localization and the extent of the lesion is of upmost importance for further treatment decisions as well as for long-term prognosis. Further studies are needed to determine the actual long-term effects of extensive defects of the lateral femoral condyle regarding early-onset osteoarthritis.

## Conclusions

In the case of a clinically suspected ACL rupture, lateral radiographs of the knee should be checked for a lateral femoral notch sign. Further MRI for confirmation of the ACL tear should also be used, carefully be inspected for the size and localization of an eventual defect to the lateral femoral condyle. The size of the lateral femoral notch may influence the prediction of the patient’s future prognosis regarding early-onset osteoarthritis, further investigations are needed to define a cut-off to support the decision between surgical and conservative treatment.

## References

[1] Graf BK, Cook DA, Desmet AA, Keene JS (1993). Bone Bruises on Magnetic-Resonance-Imaging Evaluation of Anterior Cruciate Ligament Injuries. Am J Sport Med.

[2] Prince JS, Laor T, Bean JA (2005). MRI of anterior cruciate ligament injuries and associated findings in the pediatric knee: changes with skeletal maturation. AJR Am J Roentgenol.

[3] Spindler KP, Schils JP, Bergfeld JA, Andrish JT, Weiker GG, Anderson TE, Piraino DW, Richmond BJ, Medendorp SV (1993). Prospective study of osseous, articular, and meniscal lesions in recent anterior cruciate ligament tears by magnetic resonance imaging and arthroscopy. Am J Sports Med.

[4] Murphy BJ, Smith RL, Uribe JW, Janecki CJ, Hechtman KS, Mangasarian RA (1992). Bone signal abnormalities in the posterolateral tibia and lateral femoral condyle in complete tears of the anterior cruciate ligament: a specific sign?. Radiology.

[5] Rosen MA, Jackson DW, Berger PE (1991). Occult osseous lesions documented by magnetic resonance imaging associated with anterior cruciate ligament ruptures. Arthroscopy.

[6] Sharma G, Naik VA, Pankaj A (2012). Displaced osteochondral fracture of the lateral femoral condyle associated with an acute anterior cruciate ligament avulsion fracture: a corollary of “the lateral femoral notch sign”. Knee Surg Sports Traumatol Arthrosc.

[7] Tauber M, Fox M, Koller H, Klampfer H, Resch H (2008). Arthroscopic treatment of a large lateral femoral notch in acute anterior cruciate ligament tear. Arch Orthop Trauma Surg.

[8] Warren: The lateral notch sign of anterior cruciate ligament insufficiency. American Journal of Knee Surgery 1988:119–124.

[9] Faber KJ, Dill JR, Amendola A, Thain L, Spouge A, Fowler PJ (1999). Occult osteochondral lesions after anterior cruciate ligament rupture. Six-year magnetic resonance imaging follow-up study. Am J Sports Med.

[10] Stein LN, Fischer DA, Fritts HM, Quick DC (1995). Occult osseous lesions associated with anterior cruciate ligament tears. Clin Orthop Relat Res.

[11] Nakauchi M, Kurosawa H, Kawakami A (2000). Abnormal lateral notch in knees with anterior cruciate ligament injury. J Orthop Sci.

[12] Garth WP, Wilson T (2001). Open reduction of a lateral femoral notch associated with an acute anterior cruciate ligament tear. Arthroscopy.

[13] Sadlo PA, Nebelung W (2006). Arthroscopically assisted reduction of a lateral femoral notch in acute tear of the anterior cruciate ligament. Arthroscopy.

[14] Kaplan PA, Walker CW, Kilcoyne RF, Brown DE, Tusek D, Dussault RG (1992). Occult fracture patterns of the knee associated with anterior cruciate ligament tears: assessment with MR imaging. Radiology.

[15] Speer KP, Spritzer CE, Bassett FH, Feagin JA, Garrett WE (1992). Osseous injury associated with acute tears of the anterior cruciate ligament. Am J Sports Med.

[16] Cobby MJ, Schweitzer ME, Resnick D (1992). The deep lateral femoral notch: an indirect sign of a torn anterior cruciate ligament. Radiology.

[17] Eckstein F, Benichou O, Wirth W, Nelson DR, Maschek S, Hudelmaier M, Kwoh CK, Guermazi A, Hunter D (2009). Magnetic resonance imaging-based cartilage loss in painful contralateral knees with and without radiographic joint space narrowing: Data from the Osteoarthritis Initiative. Arthritis Rheum.

[18] Graf BK, Cook DA, De Smet AA, Keene JS (1993). “Bone bruises” on magnetic resonance imaging evaluation of anterior cruciate ligament injuries. Am J Sports Med.

[19] Eckstein F, Kunz M, Schutzer M, Hudelmaier M, Jackson RD, Yu J, Eaton CB, Schneider E (2007). Two year longitudinal change and test-retest-precision of knee cartilage morphology in a pilot study for the osteoarthritis initiative. Osteoarthritis Cartilage.

[20] Hudelmaier M, Glaser C, Pfau C, Eckstein F (2012). Comparison between different implementations of the 3D FLASH sequence for knee cartilage quantification. MAGMA.

[21] Wirth W, Eckstein F (2008). A technique for regional analysis of femorotibial cartilage thickness based on quantitative magnetic resonance imaging. IEEE Trans Med Imaging.

[22] Hoffelner T, Resch H, Moroder P, Atzwanger J, Wiplinger M, Hitzl W, Tauber M (2012). No increased occurrence of osteoarthritis after anterior cruciate ligament reconstruction after isolated anterior cruciate ligament injury in athletes. Arthroscopy.

[23] Petersen WZ, T.: Indikation zur operativen oder nicht operativen Therapie der Kreuzbandruptur. In: Petersen W, Zantop T. Das vordere Kreuzband-Grundlagen und aktuelle Praxis der operativen Therapie. Köln: Deutscher ärzte-Verlag, 2009:67–76.

[24] Nakamae A, Engebretsen L, Bahr R, Krosshaug T, Ochi M (2006). Natural history of bone bruises after acute knee injury: clinical outcome and histopathological findings. Knee Surg Sports Traumatol Arthrosc.

[25] Costa-Paz M, Muscolo DL, Ayerza M, Makino A, Aponte-Tinao L (2001). Magnetic resonance imaging follow-up study of bone bruises associated with anterior cruciate ligament ruptures. Arthroscopy.

